# Functional Analysis of Gastric Tight Junction Proteins in *Xenopus laevis* Oocytes

**DOI:** 10.3390/membranes12080731

**Published:** 2022-07-23

**Authors:** Laura Stein, Nora Brunner, Salah Amasheh

**Affiliations:** Institute of Veterinary Physiology, Department of Veterinary Medicine, Freie Universität Berlin, 14163 Berlin, Germany; laura.stein@fu-berlin.de (L.S.); nora.brunner@fu-berlin.de (N.B.)

**Keywords:** claudins, epithelial barrier, *Xenopus* oocytes, tight junction, heterologous expression

## Abstract

The epithelial barrier is crucial for proper gastrointestinal function, preventing the unwanted passage of solutes and therefore representing a prerequisite for vectorial transport. Claudin-4 and claudin-18.2, two critical tight junction proteins of the gastric epithelium, seal neighboring cells in a physically and mechanically challenging environment. As the *Xenopus laevis* oocyte allows the functional and molecular analyses of claudin interaction, we have addressed the hypothesis that this interaction is not only dependent on mechanical force but also on pH. We expressed human claudin-4 and claudin-18 in *Xenopus* oocytes, and analyzed them in a two-cell model approach. Cells were clustered in pairs to form contact areas expressing CLDN18 + CLDN18, CLDN4/18 + CLDN4/18, and compared to controls, respectively. Contact areas in cells incubated in medium at pH 5.5 and 7.4 were quantified by employing transmitted light microscopy. After 24 h at pH 5.5, clustering of CLDN18 + CLDN18 and CLDN4/18 + CLDN4/18-expressing oocytes revealed a contact area reduced by 45% and 32%, compared with controls, respectively. A further approach, high-pressure impulse assay, revealed a stronger tight junction interaction at pH 5.5 in oocyte pairs expressing CLDN18 + CLDN18 or CLDN4/18 + CLDN4/18 indicating a protective role of claudin-18 for tight junction integrity during pH challenge. Thus, our current analysis of gastric tight junction proteins further establishes oocytes as an expression and two-cell screening model for tight junction integrity analysis of organ- and tissue-specific claudins by the characterization of homo- and heterophilic *trans*-interaction dependent on barrier effectors.

## 1. Introduction

Organ- and tissue-specific analysis of barrier properties have come to be a cornerstone of physiological and pathophysiological research [1,2]. In addition to tissue models, several models have recently been developed to gain further insights into cell–cell and molecule–molecule interactions, in vitro [3,4]. Tight junction (TJ) proteins were first identified as the molecular correlate of barrier function in 1993 [5], followed by the identification of the most abundant and what are considered to be the functionally most relevant family of tetraspan barrier proteins, namely claudins [6]. Today, the claudin family is known to consist of 27 members and additional splice variants, all of which show a differential expression pattern determining specific barrier properties as a prerequisite of the functional integrity in all epithelia [7].

A basic understanding regarding the functional contribution of claudins to the TJ was initially obtained from cell–cell interaction studies in cells not expressing TJ proteins, namely fibroblasts [8]. This strategy has been extended recently by establishment of the heterologous expression of claudins in *Xenopus laevis* oocytes [9]. Allowing for a variety of membrane transport analyses, the *Xenopus* oocyte has been classically employed as a heterologous expression system for analyses of membrane transporters [10]. Common analyses have included measurement of the uptake of radiolabeled substrates, and electrophysiological experiments such as voltage clamp and patch clamp approaches [11,12]. In recent years, these studies have been extended regarding analyses of gap junction and TJ proteins in paired oocyte approaches [9,13,14], which have enhanced the possibilities of single cell interaction and tissue analyses. However, the paired cell approaches do not encompass the effects of tissue factors, and thus questions remain regarding the role of endogenous effectors, including further membrane and membrane-associated proteins. One main question has been resolved just recently by the demonstration of the expression and localization of endogenous scaffolding protein ZO-1, which is known to be necessary for the cytoskeleton anchoring of integral TJ protein strands [4]. Our group has now used the momentum thus obtained for further analyses of organ-specific protein interaction, namely gastric TJ integrity.

The stomach epithelium is challenged by numerous physicochemical effectors, including food and beverages, medication and individual diets. A prerequisite for proper digestive functions, but also a potential risk factor, is the low pH value of the chyme [15]. Whereas local mucus and bicarbonate secretion are able to prevent extensive pH challenges, a contact of epithelial cells with an acidic pH cannot be fully prevented during the gastric phase and gastric cycles. Furthermore, gastric pathologies can cause proton–epithelium contact and make the epithelium vulnerable to damage from luminal acid by disruption of the mucosal barrier, e.g., the common *Helicobacter pylori* infection, a main risk factor for TJ dysfunction and gastric cancer [16].

The TJ barrier of the gastric epithelium primarily comprises claudin-18.2 (claudin-18), which has been shown to provide a barrier against H^+^. Claudin-4 and claudin-18 are critical integral membrane proteins of the TJ complex in gastric epithelium. Both claudins contribute to the barrier against the paracellular passage of cations [17,18]. A change of protein expression is associated with gastric cancer because of the dysregulation of gastric-specific barrier properties [19]. In our study, we have focused on heterologous expression of the TJ protein in *Xenopus* oocytes, testing the hypothesis that gastric claudin-18 is also able to support TJ integrity during pH challenges, namely an acidic pH. Claudin-18 (CLDN18)-expressing, and claudin-4/claudin-18 (CLDN4/18)-co-expressing *Xenopus laevis* oocytes were used as a gastric TJ protein interaction model in acidic and neutral pH values. We have therefore analyzed the pH-dependency of claudin-18 homophilic *trans*-interaction and claudin-4/18 heterophilic *trans*-interaction in vitro.

The outcome of our study provides new experimental data, showing that claudin-18 does not enhance the contact area in a paired oocyte assay, but provides a greater strength of interaction in acidic pH, which may add to the special physiological role of claudin-18 within stomach TJ strands protecting the cells and tissues from gastric acid.

## 2. Materials and Methods

### 2.1. Animals

The care and treatments of animals conformed to German legislation guidelines and was approved by the animal welfare officer for the Freie Universität Berlin and under the governance of the Berlin Veterinary Health Inspectorate (Landesamt für Gesundheit und Soziales Berlin, Germany, permit G0022/21 and O 0022/21).

### 2.2. Cloning and cRNA Preparation

For the synthesis of the human cRNAs, CLDN4 and CLDN18.2 (Lot. No. 1989156, Lot. No. 25442, Life Technologies, Carlsbad, CA, USA) nucleotide coding consensus sequences were cloned from a pMK-RQ vector into a high copy ampicillin-resistant pGEM vector via competent DH10b *Escherichia coli*. The cRNAs were produced via an in vitro T7 RNA-polymerase-based transcription system T7 RiboMAX RNA Production System and Ribo m7G Cap Analog, Promega, Walldorf, Germany) according to the manufacturer’s instructions. UV spectroscopy (P330, Implen, München, Deutschland) was used to assess cRNA concentration and purity.

### 2.3. Surgical Oocyte Harvesting and cRNA Injection

Oocytes were harvested by surgical laparotomy from female *Xenopus laevis* anesthetized in a bath solution of buffered 0.2% tricaine (ethyl 3-aminobenzoate methanesulfonate, Sigma–Aldrich, Taufkirchen, Germany) for 5–10 min at room temperature. Surgical anesthetic depth was evaluated by righting and corneal reflexes. After removing the ovarian lobes by incising the skin and abdominal muscle, oocytes were isolated by digesting connective tissue strands in 1.5 mg/mL collagenase (NB4 Standard Grade, Nordmark Pharma, Germany) and removing follicular cells by incubation in Ca^2+^-free oocyte ringer solution (ORi) on a mechanical shaker as described by Vitzthum et al. [9].

High-quality oocytes of stages V and VI (diameter > 1000 μm) were injected (Nanoliter 2010, World Precision Instruments, Sarasota, FL, USA) with 1 ng cRNA encoding human claudin-4 (CLDN4), claudin-18 (CLDN18), or with the combination of claudin-4 and -18 (CLDN4/18). Oocytes injected with RNase-free water served as controls (ctrl).

For microinjection, glass capillaries (World Precision Instruments, Berlin, Germany) were manufactured by a horizontal puller (P-97 Micropipette Puller, program 11, Sutter Instrument Company, Novato, CA, USA). For injection, oocytes were placed under a binocular in a carriage with milled grooves in a row. The injection volume was 50.6 nl per oocyte with a cRNA concentration of 20 ng/μL which corresponds to a respective cRNA amount of 1 ng per oocyte and the incubation time was 3 days at 16 °C.

### 2.4. Immunoblotting of Oocyte Membrane Fractions

For immunoblotting, membrane fractions of ten injected and pooled oocytes were prepared as described recently [4]. Oocytes were suspended in 500 μL homogenization buffer (MgCl_2_ (5 mM), NaH_2_PO_4_ (5 mM), EDTA (ethylenediaminetetraacetic acid) (1 mM), sucrose (80 mM), and Tris (Tris(hydroxymethyl) aminomethane) (20 mM); pH 7.4) and centrifuged twice at 200 rpm for 10 min at 4 °C. Cell debris was discarded and the supernatant was centrifuged at 13,000 rpm for 30 min at 4 °C to pellet cell membrane fractions. After resuspending the pellet in 80 μL homogenization buffer, colorimetric protein quantification using Pierce 600 nm Protein Assay Kit (Thermo Fisher Scientific, Hennigsdorf, Germany) was carried out.

Proteins of the membrane fraction samples were quantified in a 96-well plate and evaluated by a 562 nm plate reader (PerkinElmer EnSpire Multimode Plate Reader, Waltham, MA, USA) with bovine serum albumin Standards (Thermo Fisher Scientific, Hennigsdorf, Germany) from 125 to 200 μg/mL.

A stain-free immunoblotting kit (Stain Free TGX, Fast Cast Acrylamide, Bio-Rad, München, Deutschland) was used to control the protein transfer to the PVDF membrane. By adding 4 × Laemmli buffer (Bio-Rad Laboratories, Munich, Germany) and urea (9 mol/L, Carl Roth GmbH, Karlsruhe, Germany) as well as denaturing at 55 °C for 8 min membrane samples were prepared for immunoblotting. They were loaded onto the 10% SDS polyacrylamide gel as specified by the manufacturer. Then, 5% non-fat dry milk in Tris-buffered saline was used for PVDF membrane blocking for 120 min.

Claudin-4 and claudin-18 were detected by using specific primary antibodies (Invitrogen #32-9400, #700178, Life Technologies, Carlsbad, CA, USA) overnight at 7 °C and Peroxidase-conjugated goat anti-rabbit and anti-mouse antibodies (#7074, #7076 Cell Signaling Technology, Danvers, MA, USA) on the following day for 45 min at room temperature.

Protein signals were visualized by a ChemiDoc MP system (Bio-Rad Laboratories after adding detection solution (Clarity Western ECL Blotting Substrate, #1705061, Bio-Rad Laboratories GmbH, Munich, Germany).

### 2.5. Immunohistochemistry

For immunohistological stainings, injected oocytes were fixed overnight at 4 °C in 4% PFA (16% paraformaldehyde, E15700, Science Service, Munich, Germany).

Via a 70% ethanol to xylol gradient, oocytes were dehydrated followed by embedding cells in paraffin.

Samples were cross-sectioned (5 μm) and transferred onto slides. Before immunohistochemistry, paraffin was removed by using a xylol-ethanol gradient. Epitopes were exposed by boiling in citrate buffer (pH = 6.0). The sections were then permeabilized for 5 min at room temperature in Triton X-100 in PBS +/+ followed by an oocyte framing step using a PAP pen (Kisker Biotech GmbH & Co. KG, Steinfurt, Germany). After blocking (5% goat serum and 1% bovine serum in PBS), samples were incubated with primary antibodies raised against claudin-4 and claudin-18.2 (Invitrogen #32-9400, #700178, Life Technologies, Carlsbad, CA, USA) for 1 h at 37 °C. After four washing steps with blocking solution, secondary goat anti-rabbit Alexa Fluor-488 and goat anti-mouse Alexa Fluor-594 were added for 1 h at 37 °C. Before mounting in ProTaqs Mount (Flour Biocyc, Luckenwalde, Germany), four washing steps with blocking solution and one with distilled water were carried out. Protein signals were visualized using a Zeiss 710 confocal microscope (Zeiss, Oberkochen, Germany).

### 2.6. Paired Oocyte Assay with pH-Treatment and Contact Area Monitoring

To ensure that the oocyte plasma membranes attach to the neighboring cell, the vitelline membranes were mechanically removed with two fine forceps after placing them in mannitol to generate hypertonic shrinking of the cells.

**Figure 1:**
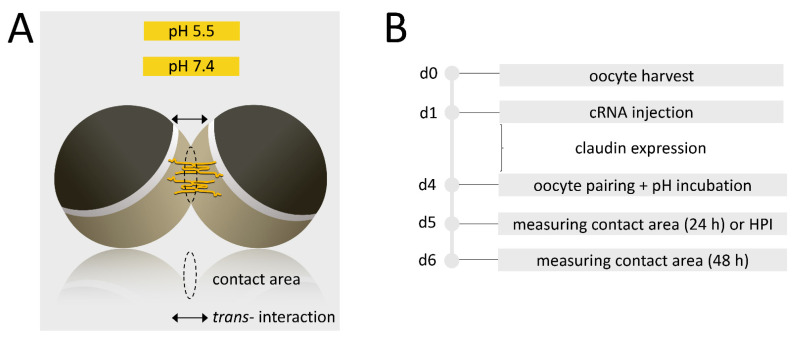
Schematic depiction (**A**) and experimental setup (**B**) of the paired oocyte assay and HPI.

Then, the paired oocyte assay was carried out with oocyte pairs of combinations of CLDN4 + CLDN4, CLDN4 + ctrl, CLDN18 + CLDN18, CLDN18 + ctrl, CLDN4/18 + CLDN4/18, CLDN4/18 + ctrl, and ctrl + ctrl. Oocytes were placed together in 24-well microliter plates by pushing them three times together using a metal probe. Analogous to that an incubation in pH 5.5 and 7.4 ORi was carried out (Figure 1). After 24 h and 48 h and storing the oocytes at 16 °C, the contact diameter via bright-field microscopy (DMI6000 B Microscope, LAS AF software Leica Microsystems, Wetzlar, Germany) was quantified. The sizes of circular contact areas (μm^2^) were calculated using the formula A = π∙r^2^. After 48 h, the condition and the cohesion of the oocyte pairs were evaluated and examined by gently pushing them apart from each other under the binocular.

### 2.7. Hydrostatic Pressure Impulse Assay and Quantification of Contact Area Strength

Oocytes were prepared for pairing and treated with pH 5.5 ORi as described before. To test the strength of the contact area, a hydrostatic pressure impulse Assay (HPI) established by Brunner et al. [14] was carried out with the oocyte combinations: CLDN18 + CLDN18 and ctrl + ctrl, such as CLDN4/18 + CLDN4/18, CLDN4/18 + ctrl, ctrl + ctrl.

Oocyte pairs were placed central to each well of a 24-well plate filled with 2 mL ORi. After 24 h a defined hydrostatic pressure impulse was applied via a single channel electronic pipette (EE-300R, Eppendorf Research Pro, software version 2.06.00, Hamburg, Germany) using a pipetting volume of 250 μL ORi (pH 5.5) at a dispensing speed of 0.9 s. The angle was 45° and the distance of application was 1.3 cm. The conditions were kept constant and oocyte pairs were subjected to the impulse in one passage.

Bright-field microscopy was used for quantifying the change of contact area before and 30 min after the hydrostatic pressure. Alterations of contact area in percentage (Δ contact area) were statistically evaluated.

### 2.8. Statistical Analysis

Data from the paired oocyte assay are expressed in means in % of the controls and standard error of the mean (SEM). Controls were set to 100%. Data from the hydrostatic impulse assay are presented as box plots, which were notched for better visualization of the median (50-percent). The first (25-percent) and the second quartile (75-percent) as well as the whiskers (10th and 90th percentile) are presented, and n is the number of oocyte pairs. JMP Pro 16.0.0 (SAS Institute Inc., Cary, NC, USA) was used for statistical analyses. Normal distribution was checked using the Shapiro–Wilk test. The Kruskal–Wallis test was performed for not normally distributed data, followed by a Dunn–Bonferroni correction. Values of *p* < 0.05 were considered to be statistically significant (presented as * *p* < 0.05, ** *p* < 0.001 and *** *p* < 0.001).

## 3. Results

### 3.1. Expression of Human Gastric Claudins in the Oocyte Membrane

**Figure 2:**
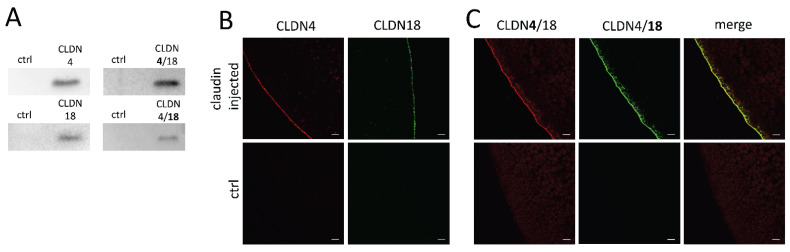
Detection of heterologous expression of claudins in the oocyte membrane by immunoblotting (**A**) and confocal laser scanning immunofluorescence microscopy (**B**,**C**). Representative images (scale bars: 10 μm).

To ensure CLDN4, CLDN18, and CLDN4/18 expression in the oocyte membrane, immunoblotting was carried out after an incubation time of 3 days. All membrane fraction samples revealed claudin-specific signals at 22 kDa (CLDN4) and 27 kDa (CLDN18) whether solely- or co-expressed, whereas RNAse-free water-injected control oocytes showed no specific signals for CLDN4, CLDN18, or CLDN4/18 expression (Figure 2A). Further, the integration of the expressed membrane proteins was visualized by confocal laser scanning immunofluorescence microscopy. Oocyte samples were stained with antibodies in accordance with the injected claudin cRNAs, and specific signals were detected, respectively. CLDN4 and CLDN18-expressing oocytes revealed specific signals in the oocyte plasma membrane (Figure 2B). Moreover, the combined expression of CLDN4 and CLDN18 revealed a colocalization of signals (Figure 2C). No signal could be detected in RNAse-free water-injected oocytes.

### 3.2. Paired Oocyte Assay Revealed pH and Claudin-Dependent Effects

**Figure 3:**
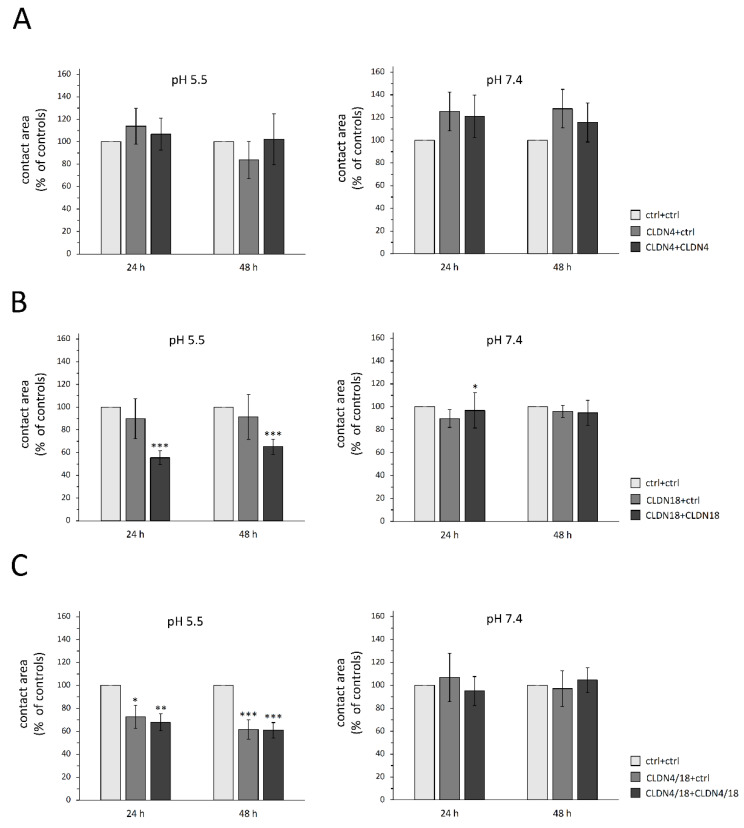
Contact areas of paired oocytes after 24 and 48 h. (**A**) CLDN4, (**B**) CLDN18-, and (**C**) CLDN4/18-expressing oocytes. Whereas no changes of contact areas were observed in oocyte pairs expressing CLDN4 alone, CLDN18 and CLDN4/18 pairs revealed a markedly reduced contact area in pH 5.5. Data are presented in mean ± SEM (n = 8–22, * *p* < 0.05 ** *p* < 0.01, *** *p* < 0.001).

To assess their *trans*-interaction areas in acidic to neutral pH within pairs expressing claudins (CLDN4, CLDN18, CLDN4/18), the contact areas of the paired cells were monitored for 24 h and 48 h. The paired oocyte assay revealed differences in contact areas depending on clustered claudin combinations and pH values. Notably, the contact area of CLDN18 and CLDN4/18-injected oocyte pairs treated with acidic pH (pH 5.5) showed a marked reduction in initial contact areas set as 100% by 45% and 32% after 24 h (CLDN18 + CLDN18: 55.5 ± 6.04%, *p* = 0.0009, n = 15; CLDN4/18 + CLDN4/18: 67.9 ± 7.42%, *p* = 0.0057, n = 16) and by 35% and 39% after 48 h (CLDN18 + CLDN18: 65.2 ± 6.64%, *p* = 0.0005, n = 15; CLDN4/18 + CLDN4/18: 60.9 ± 6.78%, *p* ≤ 0.0001, n = 16; Figure 3B,C). In addition, the clustered combination CLDN4/18 + ctrl also showed a slightly reduced contact area in pH 5.5 after 24 h compared with the ctrl + ctrl pairs (72.7 ± 10.02%, *p* = 0.0494, n = 9). This effect increased after 48 h (61.57 ± 8.54%, *p* = 0.0002, n = 9), whereas in neutral pH (7.4), all other combinations showed no effect on the contact area, except for CLDN18 + CLDN18 at 24 h (CLDN18 + CLDN18: 24 h: 92.1 ± 11.74%, *p* = 0.0367, 48 h: 94.7 ± 10.92, *p* = 0.374, n = 13; CLDN4/18 + CLDN 4/18: 24 h: 95 ± 10.81%, *p* = 0.4691, 48 h: 104.6 ± 17.19%, *p* = 0.32, n = 22). None of the contact areas of CLDN4-expressing clustered oocytes showed significant differences in acidic or neutral pH (pH 5.5: 24 h: *p* = 0.9584, 48 h: *p* = 0.4079, n = 8–11; pH 7.4: 24 h: *p* = 0.7602; 48 h: *p* = 0.7686, n = 9–14; Figure 3A).

### 3.3. Hydrostatic Pressure Impulse Assay and Quantification of Contact Area Strength

**Figure 4:**
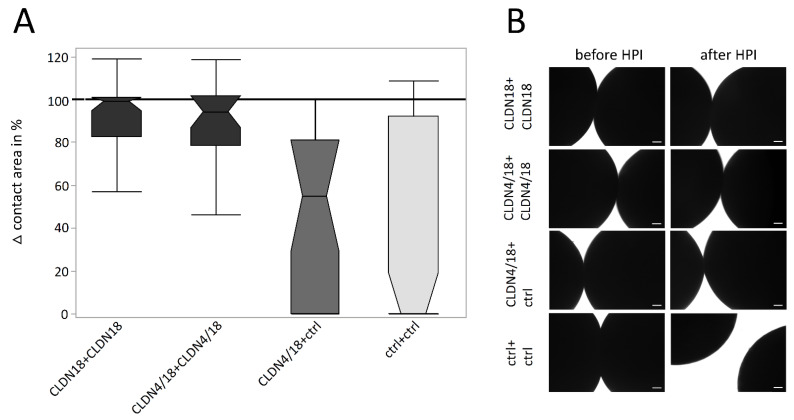
Change of contact areas before and after HPI in pH 5.5 (**A**) and its visualization and quantification by transmission electron microscopy (**B**). Whereas the combination of oocytes expressing CLDN18 + CLDN18, and CLDN4/18 + CLDN4/18 maintained a larger contact area vs. ctrl after HPI. The difference between CLDN4/18 + ctrl and ctrl pairs was not significantly altered (n = 25–60, Kruskal–Wallis test followed by a Dunn–Bonferroni correction. Representative images; scale bars = 100 μm).

To analyze the strength of oocyte pair attachment, the combinations with decreased contact areas, CLDN18 + CLDN18, CLDN4/18 + CLDN4/18, and CLDN4/18 + ctrl in acidic pH (pH 5.5), where challenged by a hydrostatic pressure impulse assay (HPI). After HPI, clustered CLDN4/18 and CLDN18-expressing oocytes lost approximately 20% of the contact area (CLDN18 + CLDN18: 81.03 ± 6.03%, *p* ≤ 0.0001, n = 38; CLDN4/18 + CLDN4/18: 82.19 ± 6.85%, *p* = 0.0001, n = 25). The median was close to 100% (99% and 94%), indicating that the hydrostatic pressure reduced the contact area only slightly (Figure 4A,B). In contrast, contact areas of the ctr + ctrl combination decreased by 60% (ctrl + ctrl: 39.61 ± 8.22%, n = 60). The median was 0%, because most of the pairs separated due to the pressure. Moreover, the HPI assay values of CLDN4/18 + ctrl and ctrl + ctrl pairs were not significantly different (42,98% ± 8.22, *p* = 1.000, n = 25; Figure 4A). The median of the CLDN4/18 + ctrl group was 54%, implying that contact areas were largely decreased or loosened.

## 4. Discussion

Gastrointestinal barrier integrity represents a basic physiological requirement for individual body function. Paired oocyte assays have been carried out from the time at which connexin was identified and functionally analyzed [20]. The prerequisite for the currently applied paired oocyte assay was the expression of CLDN4, CLDN4/18, and CLDN18 after three days of injection of the cRNA. The results demonstrated a successful heterologous expression at the correct size. In addition, claudins were localized in the oocyte membrane using immunohistochemistry. The immunoblots and confocal immunofluorescent stainings confirm the *Xenopus* oocyte as an appropriate heterologous expression system also for the gastric TJ proteins claudin-4 and claudin-18, after other human TJ proteins claudin-1, -2, -3, and -5 have already been successfully heterologously expressed and characterized in oocytes by our group [4,9] and other expression models, namely epithelial cells [21,22], respectively.

Claudin-18 has two isoforms: the lung-specific claudin18.1, and claudin18.2, which is primarily expressed in stomach epithelia [23]. However, much is unknown regarding the assembly of claudin-18 into gastric TJs, its individual functions, and its contribution to the barrier, and only a few studies analyzed the role of claudin18.2 in knockout models. The deletion of claudin-18 resulted in an increased paracellular H**^+^** leakage into the submucosa caused by the loss of parallel packed TJ strands [17,18,24]. Furthermore, claudin-18 is expressed in small amounts in duodenum and abundantly expressed in Barret’s esophagus, where it contributes to a better epithelial acid resistance [25,26]. Thus, claudin-18 forms closely anastomosing TJ strands and contributes to the paracellular barrier against luminal protons from luminal acid.

The loss of claudin-18 not only results in TJ dysfunction and lack of proton barrier properties but also leads to numerous pathologies including gastritis, chronic inflammation, and gastric cancer, therefore claudin-18 has been identified as a targeted therapy candidate [18,27,28,29,30]. The goal of our study was the analysis of the functional contribution of the gastric TJ protein claudin-18.2 in the presence and absence of co-expressed claudin-4 based on a two-cell model assay, which enables the creation of a deliberated contact area for cell–cell interaction or rather head-to-head claudin interaction.

Part of the two-cell model was the pH- and claudin-dependent analysis of contact areas via the paired oocyte assay, and the investigation of the interaction in *trans*, namely, the intercellular interaction of claudins via extracellular loops, in the hydrostatic pressure experiment, as *trans*-interactions are the basis for claudin cluster assembly and TJ strand formation [31,32,33].

The paired oocyte assay revealed significantly smaller contact areas for CLDN18-expressing and CLDN4/18-co-expressing oocyte pairs in acidic pH, whereas in neutral pH, apart from CLDN18 + CLDN18 pairs, which showed a slightly reduced contact area, no effects were observed. The challenge of the contact area of these combinations by hydrostatic pressure in pH 5.5 revealed stronger adhesion in accordance with *trans*-interactive properties. In summary, the claudin-18 contact area was not increased in the paired oocyte assay after 24 h and 48 h but showed a stronger interaction in the hydrostatic pressure experiment at acidic pH when solely expressed or co-expressed with claudin-4. This may be due to its function of protecting cells and tissues from gastric acid. These results underline the function of claudin-18 in protecting vulnerable epithelium by blocking the paracellular permeability of cations, especially H**^+^** [16,17,24], via the formation of stronger cell–cell contacts in the acidic environment.

Thus, the pH value as an important luminal gastrointestinal parameter as well as the claudin composition affects cell–cell interaction, especially to the *trans*-interaction of CLDN18 and CLDN4/18 in size and strength, which highlights the notion that claudin-18 seems to have a significant physiological role in the specific gastric environment.

It should be noted that the CLDN4/18 + ctrl clustering had a similar effect on the contact area size after 48 h, but the strength after 24 h between CLDN4/18 + ctrl and ctrl + ctrl pairs was not significantly altered. Because of the co-expression of claudin-4 and claudin-18 in one cell, heterologous *cis*-interactions between the claudins were possible. This assumption is also supported by the immunohistochemical stainings, which revealed colocalization of claudin-4 and claudin-18 signals. In the literature, *cis*-interactions were also described also as oligomerization, yet information regarding heterodimeric oligomers consisting of claudin-4 and claudin-18 is scarce [34]. For the time being, a direct quantitative comparison of claudins is challenging, but further techniques such as freeze fracture electron microscopy might shed more light on these comparisons in the future.

Moreover, the kinetics of TJ assembling and scaffolding are provided by the cytoplasmatic protein ZO-1 by connecting cytoskeletal actin filaments and TJ proteins such as claudins [35,36]. The literature demonstrates that ZO-1 exists in *Xenopus* embryos from the first cleavage stage on and according to this, our group detected the endogenous expression of α+ and α− isoforms of ZO-1 in stage V and VI oocytes [4,37]. Thus, a stable incorporation and homophilic or heterophilic interaction of heterologous CLDN4 and CLDN18 are enabled by the endogenous expression of ZO-1 in the *Xenopus* oocyte.

Extension of the heterologous *Xenopus laevis* oocyte expression system to analyses of gastric TJ proteins might now enable further physiological studies, e.g., to analyze the effects of secondary plant compounds such as alkaloids, flavonoids and phenolic acids. Protective, e.g., antiulcerogenic activity of secondary plant compounds could be caused by enhancement of the gastric antioxidant defense system, but also due to direct barrier strengthening effects [38,39,40], whereas adverse effects may also be possibly detectable [41]. The heterologous expression of human gastric-specific claudins in the *Xenopus laevis* oocyte might allow a simple and cost-efficient examination of some of these mechanisms. Our current study provides new possibilities for TJ integrity and gastric claudin *trans*-interaction analysis dependent on extracellular factors.

## Data Availability

Data is contained within the article. The datasets analyzed during the current study are available from the corresponding author upon reasonable request.
